# Role of peripheral immune cells in spinal cord injury

**DOI:** 10.1007/s00018-022-04644-0

**Published:** 2022-12-07

**Authors:** Cong Li, Wu Xiong, Bowen Wan, Guang Kong, Siming Wang, Yingying Wang, Jin Fan

**Affiliations:** 1 Department of Orthopedics, The First Affiliated Hospital of Nanjing Medical University, Nanjing, 210029 China; 2 Department of Orthopaedics, Subei People’s Hospital of Jiangsu, Clinical Medical College of Yangzhou University, Yangzhou, China; 3 Nanjing Medical University, Nanjing, 210029 China; 4 State Key Laboratory of Natural Medicines, Department of Pharmaceutical Science, China Pharmaceutical University, Nanjing, 210009 China

**Keywords:** Spinal cord injury, Immune system, Inflammation

## Abstract

Secondary spinal cord injury is caused by an inflammatory response cascade, and the process is irreversible. The immune system, as a mediator of inflammation, plays an important role in spinal cord injury. The spinal cord retains its immune privilege in a physiological state. Hence, elucidating the mechanisms by which peripheral immune cells are recruited to the lesion site and function after spinal cord injury is meaningful for the exploration of clinical therapeutic targets. In this review, we provide an overview of the multifaceted roles of peripheral immune cells in spinal cord injury.

## Introduction

With the continuous progress of human civilization and the gradual improvement of medical and health technology, the incidence rate of spinal cord injury has risen sharply (the annual incidence rate is 60/ million) and is showing a younger trend. Spinal cord injury (SCI) is usually secondary to spinal fractures caused by direct or indirect external forces, such as traffic accidents and falls from high altitudes [1]. It often causes severe limb dysfunction below the injured segment or even death. This problem is receiving increased attention, for serious injury caused by its initial injury, and the complicated pathogenesis caused by its secondary injury. If not addressed promptly, the cascade amplification reaction will cause irreparable harm to patients. The pathogenesis of spinal cord injury has always been a hot topic because it can guide the precise localization of our clinical treatment. Centering on this problem and combining with existing research, we found that this is a multi-cell, tissue, and system interaction comprehensive disease. Therefore, we should analyze the problem and eliminate the limitation comprehensively from many angles. After the destruction of the blood–spinal cord barrier, the immune privileges of the central nervous system break down. As we know, secondary spinal cord injury is mainly caused by an inflammatory cascade response, and the immune system plays a critical role in inflammation regulation [2]. According to a previous study, the appearance of peripheral immune cells in the injured spinal cord confirmed that immune system medicates the secondary spinal cord injury. The review will focus on this part to discuss the impact of infiltration of peripheral immune cells after a spinal cord injury.

### Destruction of the blood–spinal cord barrier and invasion of peripheral immune cells

**Fig. 1:**
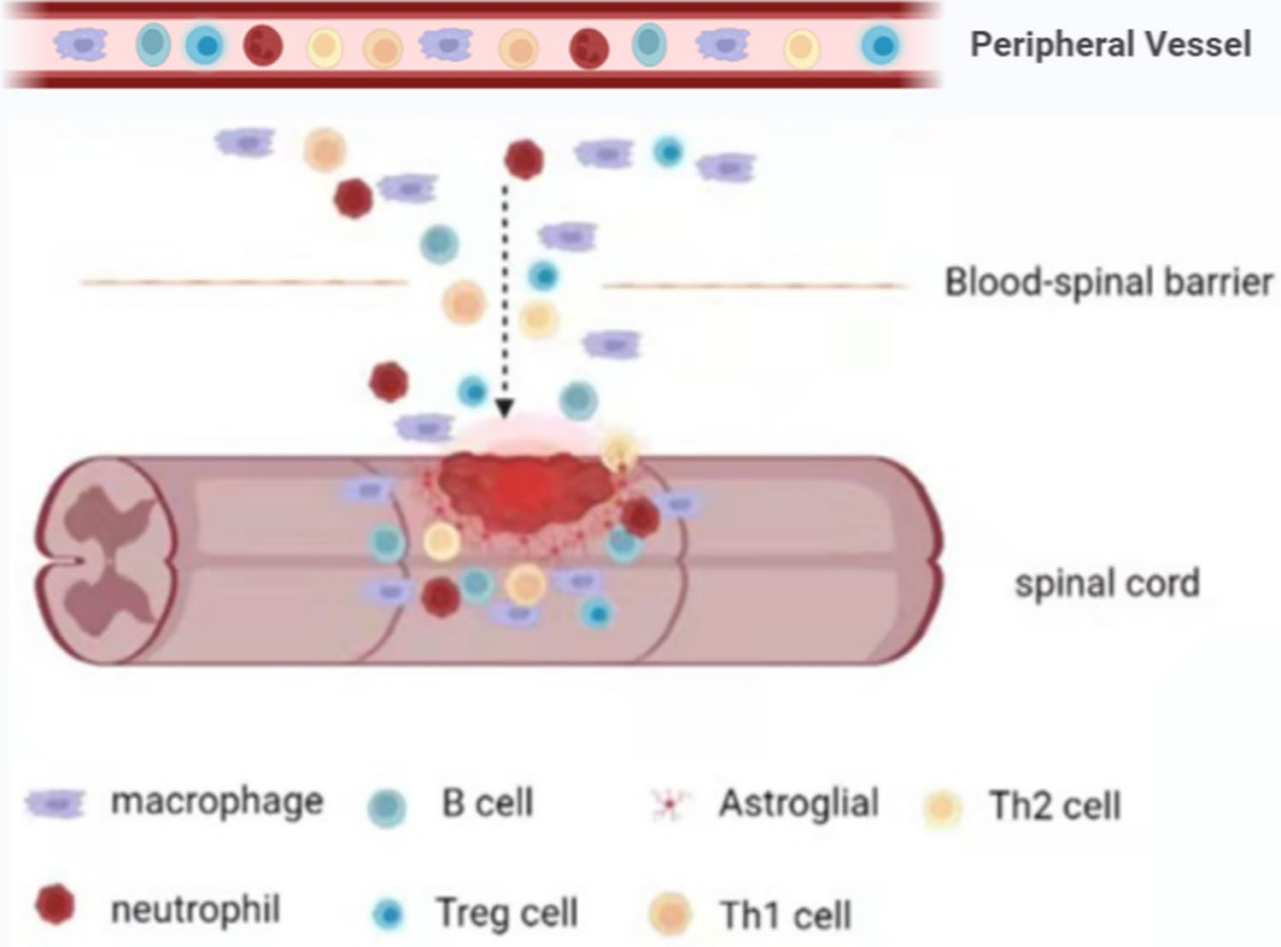
The destruction of the blood–spinal cord barrier: primary damage broke the blood–spinal cord barrier and deprived the immune privilege. Demyelination or cell death released a large number of inflammatory cytokines and chemokines that recruited immune cells from the peripheral blood circulation to the injured site. Neutrophils, macrophages, and lymphocytes migrated to the injured cord and functioned

The blood–spinal cord barrier (BSCB) consists of the tight junction (TJ) protein connecting adjoining capillary endothelial cells, which act as physical barriers that block macromolecular substances from entering the central nervous system (CNS) and prevent autoimmune diseases. It is important in maintaining a stable and typical neurological function in the spinal cord bio-environment [3]. However, after compression injury of the spine, the integrity of BSCB is destroyed, leading to infiltration of peripheral immune cells to the injury site. The destruction is a crucial step in the escalation of secondary SCI as it triggers the mobilization of inflammatory cytokines across the injured area (Fig. 1). More evidences show that the damage of the BSCB is a prerequisite for immune cells to enter the injured site and has a negative effect on the prognosis of SCI [4].

**Fig. 2:**
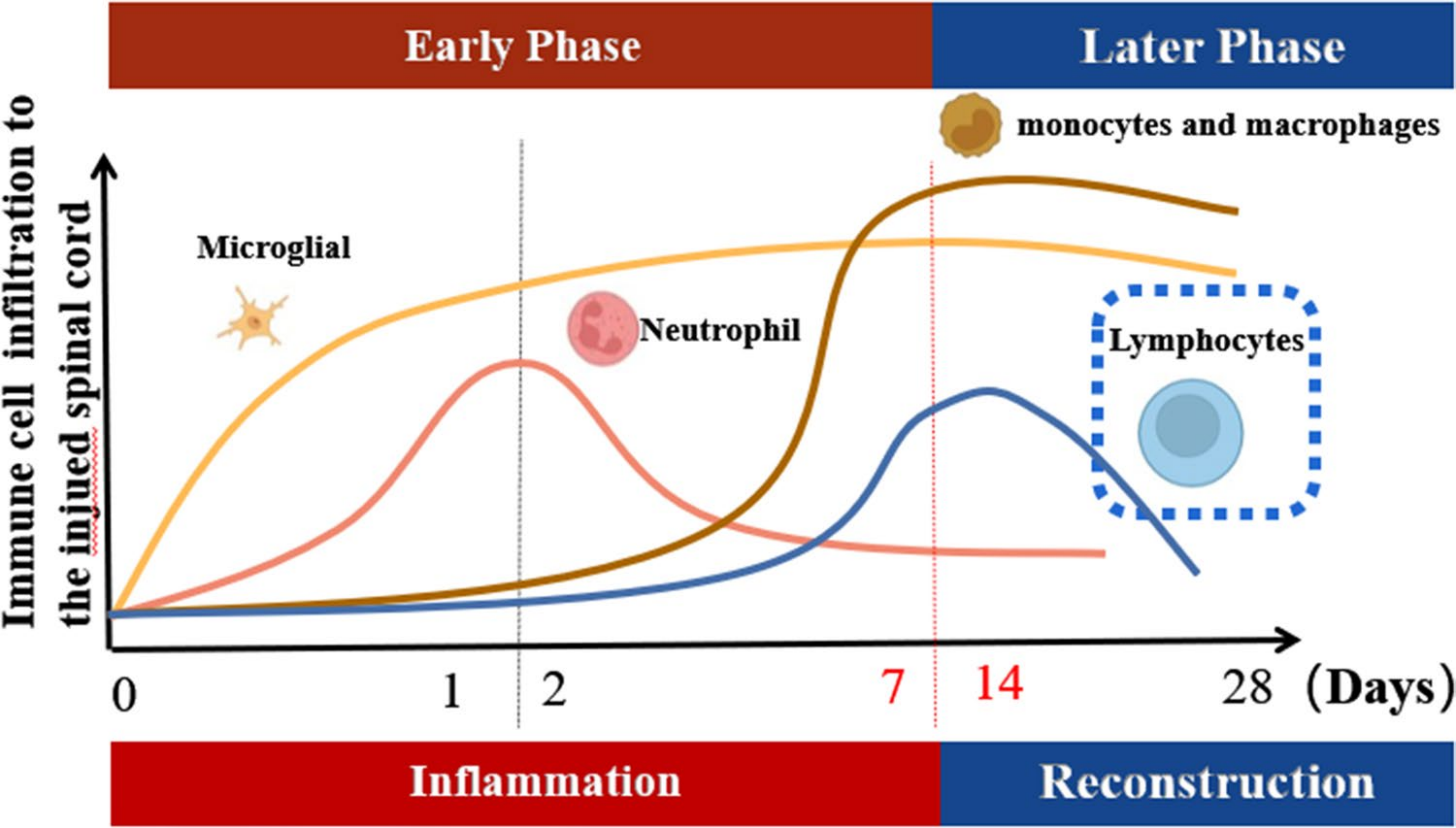
Time window of immune cell infiltration: granulocytes entered the injury site 6–12 h after SCI, reached a peak at 24 h and continued to decrease after 48 h. The migration of monocytes began about 3 days after injury and reached a peak after about 7 days. Lymphocyte aggregation mainly occurred 7 days after injury, and had a stable decline period in the subsequent 2 weeks

The initial damage disrupts the integrity of the neurotransmitter system, releasing large amounts of myelin sheath debris and activating local inflammation. Furthermore, the immune cascade after SCI involves a positive feedback process that secretes chemokines such as CXCL10 and CCL-2 which promote peripheral immune cell infiltration to the lesion site [5]. Although the specific mechanism is complex and largely unclear, a recent report showed that the complicated effect of immune cell migration could play a negative role in the functional recovery of the spinal cord and regeneration of neurons. Additionally, the early immune inflammatory event after spinal cord injury involves the sequential mobilization of three main types of peripheral immune cells, i.e., 1) neutrophils as the first immune inflammatory cells to reach the injured site after which infiltration reaches a peak 24 h after injury; 2) macrophages are subsequently recruited from the circulatory system (they reach a peak 7 days after injury). They then release inflammatory components such as the tumor necrosis factor-α (TNF-α), interleukin-1β(IL-1β), leukotrienes, nitric oxide(NO) as well as prostaglandins; 3) lymphocytes which invade the injured site by the secreted cytokines of macrophages, participate in immune inflammation [6, 7]. Notably, the time at which each cell begins to infiltrate the injured site and the period of cell decay were reported to be different making their windows in SCI not to coincide completely [8–10] (Fig. 2). This, in turn, promotes mutual chemotaxis and activation of the immune cells. In fact, a preliminary consensus was achieved with regard to the function of immunity as well as inflammation in diseases of the nervous system, especially in SCI. Nonetheless, the specific role of each type of immune cell as well as more detailed molecular and cellular mechanisms are still unclear.

### Neutrophils

Neutrophil secretion arises from the hematopoietic cord located in the venous sinuses of the bone marrow and originates from a universal committed myeloid progenitor. Notably, G-CSF is the primary modulator of this process and its roles include the engagement of progenitor cells to myeloid origin [11], multiplication of neutrophil precursors, reducing the time of subdivision, and secretion of mature neutrophils from the bone marrow [12]. Furthermore, other signaling pathways such as CXCL12–CXCR4 or CXCL1/2–CXCR2 additionally steer the recruitment and induction of neutrophils under inflammatory conditions. The surface expression of granulocytes identifies with its biological function. CD66b and CD11b/c can mediate cell–cell adhesiveness and interaction, as well as CD13, CD16 or CD88 (among others) which are responsible for distinct attributes of the immune response. Additionally, some chemokines that possess a glutamate–leucine–arginine motif before the amino-terminal CXC motif (ELR-CXC) have a pivotal role in neutrophil induction [13]. Particularly, the ELR-CXC chemokines, which contribute to neutrophil stimulation through the CXCR2 signal, cover CXCL8, CXCL1, CXCL5, and CXCL2 [14]. It triggers a change in the expression of integrin on the cell surface from low affinity to high affinity [15], enabling it to bind strongly to a ligand (immunoglobulin-like cell adhesion molecule, ICAM).

Neutrophils are crucial given that they are the first immune cells to infiltrate the injured spinal cord and attain the highest numbers within 24 h but decline equally as fast within the first 7 days [6, 16, 17]. The means through which neutrophils are mobilized to the injured tissue were classified. For example, it was observed that blocking the leukotriene B4/BLT1 receptor signaling or inhibition of phosphodiesterase 4 [18] and myeloperoxidase could significantly reduce the number of infiltrated neutrophils [19]. Moreover, CXCL1 was considered as a neutrophil chemokine produced by spinal cord astrocytes through the activity of IL-1 receptor (IL-1R)/MyD88 signal [20]. In addition, the concentration of CXCL1 in the serum of SCI patients was reported to escalate within the first week of injury relative to healthy subjects [21]. Existing evidence also suggests that the blockade of the repressor of the subunit β (IKKβ) of NF-κB kinase diminishes the production of CXCL1 and the subsequent neutrophil invasion, as well as proinflammatory gene expression, concurrently enhancing tissue maintenance and motor function. It suggests the prominent function of the NF-κB signaling cascade, in both neutrophil infiltration and bioactivity in the lesion. Besides, previous research showed that diminishing neutrophil invasion enhances regeneration in both rat and mouse SCI models. This is suggested to be due to the binding of neutrophils to multiple adhesive biomolecules such as VCAM-1, expressed on the inflamed endothelium when entering the injured tissue.

The specific role of neutrophils in the SCI model remains unclear. They perform bactericidal functions through phagocytosis and clearance of debris. Meanwhile, they are also considered to be indicators of a toxic tissue environment since their infiltration and accumulation at the inflammatory core of injured tissues trigger them to produce proteases, oxidative as well as tissue-degenerating enzymes (including matrix metalloproteinase 9 and TNF-α). Activation of these molecules, in turn, promotes neurotoxicity in neurons [22]. Additionally, the neutrophil–neuron cell contact seems to give rise to cytotoxicity [23]. Moreover, it was observed that a decrease in the accumulation of neutrophils at the lesion site could lead to decreased expression of proinflammatory cytokines, apoptosis, oxidative stress, and a remarkable elevation of motor regeneration in most conditions. Although it is known that neutrophils serve an unfavorable role in the inflammatory reaction, their function in the regeneration processes needs to be explored in detail. Furthermore, increasing evidence shows that neutrophils confer an indirect advantageous effect by initiating inflammation-associated tissue repair. For instance, a recent study for the first time demonstrated the relationship between the presence of neutrophils and reduction in the levels of reactive oxygen species (ROS) at the injured site using particular antibody-triggered approaches of Ly6G/Gr-1 + neutrophil exhaustion [24]. This revealed the importance of neutrophils in the moderation of inflammatory responses and successive regeneration of tissues following SCI [25]. Additionally, a previous study suggested that neutrophils can decrease inflammation and promote axon regeneration by secreting the secreted leukocyte protease inhibitor (SLPI), which is essential for SCI regeneration, highlighting the positive role of neutrophils [26].

### Macrophages

Macrophages are distributed throughout the body and can be broadly divided into tissue and circulating macrophages. Under normal physiological state, they oversee the pathology of the tissue bio-environment, keep tissues in a steady state, phagocytose dead and dying cells as well as react promptly to disturbances in the local surroundings. Following the apoptosis of neutrophils, chemical signals such as MCP-1 and CCL2 recruit macrophages to the injured site. Meanwhile, other inducers, including chemokines such as MIP-1a, CCL3, and IL-1β have been reported to be effective in recruiting peripheral macrophages to eliminate apoptotic neutrophils [27]. Additionally, they are the main effector cells of the innate immune response during SCI. However, conflicting reports exist on the role of macrophages in SCI. For instance, some studies suggested that macrophage infiltration and the associated inflammatory response were involved in secondary tissue damage and injury during SCI. Nonetheless, other reports propose that they play a positive role in tissue protection and repair during SCI. These contradictory suggestions may be because macrophages express different phenotypes in response to various stimuli resulting to disparate functions at all phases of inflammation. Moreover, the phenotypes are not fixed. In the inflammatory stage and repair stage after injury, the phenotypes of macrophages may be mutually convertible, which also makes their roles in secondary SCI more diverse and important [28]. Classical concepts divide macrophages into the M1 type characterized by iNOS activity and the M2 type characterized by Arg activity [29]. However, several studies have shown that there may be a sequence of phenotypes between the M1 and M2 macrophages in vivo after SCI. Although there is no definite marker to distinguish the M1/M2 phenotype of macrophages, they can roughly be distinguished by the different stimuli they receive and later by the secreted cytokines. In general, the T helper 1(Th1) cell-derived supernatant that is enriched in IFN-γ, TNF-α as well as IL-2 triggers M1 ‘classical’ polarization. On the contrary, the T helper 2(Th2) cells and regulatory T(Treg) cells that produce IL-4 divert macrophage ‘alternatively activated’ polarization toward the M2 type. Moreover, M1 macrophages secrete high doses of the proinflammatory cytokines IL-1, TNF-α, and IL-6 in the early stage of inflammation. On the other hand, M2 macrophages express high levels of arginase -1, IL-10, CD206, and TGF-β at the proliferative and reconstruction phase [30]. Furthermore, the condition of macrophages in vivo is much more complex compared that in vitro and may constitute an array of distinct but overlapping functional phenotypes.

Macrophage polarization after SCI usually requires remarkable changes in gene expression modulated by transcription factors. In addition, INF-γ and LPS are the typical activation ligands for stimulating M1 polarization. Generally, INF-γ can bind to the INF-γ receptor and promote M1 polarization through the STAT1 signaling cascade. However, given that INF-γ is not excessively expressed at the SCI site [31], it is not known if this signaling cascade plays an important role in the polarization of M1 macrophages after SCI. Moreover, although LPS induction does not directly mediate sterile inflammation after SCI, its receptor TLR4, one of the main receptors of injury-related molecular patterns (DAMPs), exists widely in the injured site [32] and is highly expressed in the plasma samples of patients with SCI [33]. The TLR4 receptor can signal by activating NF-κB, which is a typical transcription factor for multiple proinflammatory cytokines containing IL-6, TNF, IL-1β, and COX2, and it is also an efficient inducer of M1 polarization [34]. Additionally, both TNF and IL-1β are used in the sterile inflammation model of M1 polarization in vitro and both cytokines are excessively expressed after SCI. According to a previous study, the peak value of TNF gene expression was observed in the first hour after injury, while that of IL-1β appeared about 12 h following injury [35]. In addition, TNF activates TNFR1, which eventually results in activation of the NF-κB pathway and polarization of M1 macrophages. Furthermore, it was reported that TNF signal inhibitors could improve the regeneration of motor function following SCI although it is not known whether this effect is induced by the influence on macrophage polarization [36]. Besides, IL-1β initiates the NF-κB pathway by binding to IL-1R, which leads to polarization of M1 macrophages. Previous experimental results showed that deletion of the IL-1β gene had a positive influence on prognosis in mice [37]. Additionally, IL-4 and IL-10 are classical polarized ligands of M2 macrophages. IL-4 activates STAT6 after binding to IL-4Ra and STAT6 which, in turn, plays an important role as a vital modulator of the M2 phenotype. STAT6 can also stimulate the induction of other transcription factors that promote M2 polarization including PPARγ, KLF4, and PPARδ [38]. Moreover, stimulation of PPARγ results in the expression of typical M2-related genes, i.e., Arg-1 and MMR (CD206). On the other hand, a combination of IL-10 and IL-10R initiates the JAK1/STAT3 cascade, which indirectly inhibits the release of proinflammatory cytokines by increasing the expression of diverse effector genes [39]. Notably, after SCI, IL-4, IL-10, and most other anti-inflammatory cytokines are expressed acutely and transiently [40], they lead to the long-term existence of M1 macrophages in the injured site.

Macrophages at the injured site can regulate the clearance of cell debris through surface receptors. After spinal cord injury, most of the cell debris comes from myelin and clearance mainly depends on CR3 (Mac-1, CD11b), SR-AI/II (Msr1), and FcR [41].However, since no myelin antibody was found in the injured site after SCI, it is possible that the macrophage FcR pathway is not dominant in spinal cord injury. Additionally, injection of purified myelin into the spinal cord of mice caused the infiltration of a large number of neutrophils as well as macrophages and increased the expression of many proinflammatory cytokines although these effects were markedly reduced in CR3 silenced mice. This reveals that CR3 is a crucial biomechanism of myelin phagocytosis in the spinal cord [42]. Nonetheless, existing studies have shown that CR3 knockout in mice reduces inflammation levels and improves function, but there are doubts about whether it works by lowering foam cell levels. Our previous studies confirmed that MSR1 can mediate secondary injury after SCI by promoting the formation of foamy macrophages. In addition, macrophage MSR1 enhanced the secretion of inflammatory cytokines by stimulating the NF-κB signaling cascade, resulting in apoptosis of the neurons [43]. The Class b scavenger receptor CD36 (SR-B2) was also proven to participate in myelin phagocytosis after spinal cord injury [44]. CD36 can generate multi-receptor complexes with toll-like receptors and regulate the inflammatory phenotype of macrophages by endocytosis of the complexes or inducing specific intracellular signal cascades (including the upregulation of PPAR transcription) [45]. It is worth noting that although deletion of the CD36 gene only leads to a moderate decrease in the amount of macrophage lipid droplets in at the injured site, it significantly improves the range of injured tissues and the recovery of exercise ability [44]. This emphasizes the importance of studying macrophage function and other receptors in the microenvironment of spinal cord injury. Apart from the classical myelin receptor, clearance of myelin fragments by macrophages involves many other receptors. For instance, collectin placenta 1 (CL-P1), the scavenger receptor A (SCARA4) upregulated in multiple sclerosis and the tyrosine kinase phagocytosis receptor (MerTK) of the TAM family as a drug inhibitor, were shown to reduce uptake of the myelin sheath by macrophages in vitro [46, 47]. Moreover, adiponectin (an agonist of the adiponectin receptor) and other ligands can also enhance lipid outflow and reduce the production of proinflammatory cytokines by inducing the PPAR/LXRα/ABCA1 pathway. This, in turn, restores the normal function of macrophages and reduces the formation of myelin foam cells [48]. However, most studies on receptors are verified by in vitro experiments, and the role of these receptors in animal models of spinal cord injury is still unclear.

Macrophages can also interact with microglia residing in the central nervous system. It was observed that the beginning of macrophage infiltration was related to decreased phagocytosis of microglia, which supports the above point. The co-culture of macrophages and microglia isolated from injured areas resulted in a decline in the expression of inflammatory cytokines, including IL-1β, which may have been caused by the inhibitory signal emitted by prostaglandin E2 after binding with the EP2 receptor. Enhanced microglia stimulation and regeneration of defective motor function after SCI in CCR2-deficient mice further confirmed the anti-inflammatory effect of macrophages following SCI [49]. Macrophages can also improve spinal cord recovery by moderating tissue remodeling. It was reported that injecting a new polyphosphazene hydrogel loaded with M2 macrophages into the injured area of rats with SCI almost eliminated the cavity of the injured site, significantly improved tissue retention, promoted the infiltration of fibroblasts around blood vessels, and remodeled the extracellular matrix. Additionally, it enhanced axon growth and motor recovery in the rats [50]. On the contrary, eliminating macrophage infiltration through the administration of minocycline or eliminating fibrosis through the administration of paclitaxel all lead to cavitation at the injured site [51]. However, extensive evidence also shows that macrophages are harmful to spinal cord repair and regeneration. Many macrophage exhaustion/ablation models have shown functional recovery and improved histological morphology, indicating that macrophages are neurotoxic and hinder regeneration [52, 53]. One possibility of this functional improvement is the decrease in fibrotic scar caused by the decrease in macrophages [52].

All in all, these positive or negative effects of macrophages on cell rejuvenation are primarily attributed to their distinct polarization conditions (i.e., M1 type or M2 type). Apart from the mentioned stimulation, the manner in which they enter the CNS at the time of injury may also affect the polarization state. It was shown that repair and neuroprotective macrophages originated from the choroid plexus and reached the injured site through the central tube, while more inflammatory macrophages from hematopoiesis entered through the blood–spinal cord barrier [54]. Nonetheless, the various impacts are not necessarily due to the different subgroups. For example, yeast polysaccharide activated macrophages were proven to simultaneously have harmful and recovery effects in the spinal cord, indicating that these reactions can occur concurrently within the same macrophage subset [55]. Therefore, the point is not to entirely eliminate inflammation but to ensure an effective and appropriate synchronization of phenotypic transformation, for more conducive regeneration at an appropriate time.

### T cells

T lymphocytes originate from bone marrow progenitors whose maturation and selection occur in the thymus. They are then exported to blood circulation and migrate into peripheral immune tissues. T cells can be divided into the following phases; (1) naive or resting, (2) effector or activated, and (3) memory T cells [56]. The activation and metabolism of T cells are jointly managed by three distinct signals for promoting rapid cell growth and proliferation. These include the T cell receptor (TCR) which offers antigen specificity, costimulatory receptors, supplied by induced antigen-presenting cells (APCs) and cytokines that facilitate the growth as well as differentiation of lymphocytes [57]. Including phosphatidylinositol 3-kinase (PI3K)/protein kinase B (Akt), mammalian target of rapamycin (mTOR), metabolic kinase, AMP-activated protein kinase (AMPK), the cytokines modulate the expression and bioactivity of transcription modulatory factors including the bone marrow tumor oncogene (Myc) and the hypoxia-inducible factor-1α (HIF-1α) [58]. Immune responses commence when naive T cells encounter antigens and costimulatory ligands presented by dendritic cells (DC) [56]. In response to the different antigens encountered, the naive T cells proliferate, grow, and differentiate into distinct sub-clusters. Activated T cell can be divided into CD4^+^ T cells and CD8^+^ T cells. Activated CD4^+^ helper T cells (Th) are further grouped into four distinct sub-clusters. They include type-1 (Th1), type-2 (Th2), type-17 (Th17), and regulatory T cells (Tregs), each of which is unique with regard to function and production of cytokines [59]. On the other hand, activated CD8^+^ T cells differentiate into cytolytic T cells (CTLs) characterized by the secretion of granzyme B, TNF-α, IFN-γ, and perforin, hence contributing to the depletion of pathogens [60]. Moreover, the gradual recession of immune inflammatory reactions mediates the programmed death of activated T cells and only a proportion of the primary T cell population survives to mature into memory T cells [61].

Th cells are the primary drivers of the neuroinflammatory response. Th1 cells, characterized by the transcription factor T-bet, mainly release IFN-γ, TNF-α as well as IL-2 and mount a defense against viruses, mycobacteria, and protozoa by facilitating the stimulation of macrophages and accelerating the removal of bacteria. Th2 cells are characterized by the transcription factor GATA3. They release IL-4, IL-5, IL-9 as well as IL-13 and provide protection against extracellular parasitic infections. Th17 cells, characterized by the transcription factor ROR-γt, mediate the secretion of IL-17, IL-21, and IL-22 [62]. Differentiated CD4^+^Th1/Th2 cell lines have polarized cytokines and anti-regulation ability, which is a typical example of hosts’ response to pathogens and establishment of a memory response. Adaptive immunity was reported to be biased toward the Th1 proinflammatory phenotype after SCI [63]. One research found that Immune deficiency in mice with SCID was shown to lead to better regeneration in motor function. It is possible that the inflammatory pathway mediated by proinflammatory cytokines produced by Th1 cells contribute to secondary SCI and neurotoxicity. Combined with the existing data, we speculate that the secreted cytokines may work through JAK-STAT PI3K/Akt/mTOR and NF-κB or act on their specific receptors. Additionally, they may regulate the activation and polarization of subsequent T cells through the TCR–MHC recognition and binding system [64, 65]. Meanwhile, Th1 cells can facilitate the activation of CD8^+^CTL cells by enhancing the expression of IL-2. It was previously proven that CTL cells aggravate the destruction of the blood–spinal cord barrier and degeneration of neurons/myelin through the GrB/perforin pathways. This, in turn, promotes stimulation of the caspase-3/Poly ADP ribose polymerase (PARP) cascades, which results in neuronal apoptosis. Moreover, destruction of the blood–spinal cord barrier amplifies the immune cascade response and allows for the entrance of peripheral immune cells, including macrophages and neutrophils.

Naturally existing Foxp3^+^CD25^+^CD4^+^ regulatory T (Treg) cells undertake the role of monitoring the preservation of immunological self-tolerance and homeostasis. Tregs can be split into two sub-classes: the natural regulatory T cells (nTreg) and the induced regulatory T cells (iTreg). The two subsets are distinguished by their origins and gene expression as well as biological characteristics [66]. nTregs stem from the thymus and their maturation as well as proliferation is regulated by the thymus microenvironment after exposure to the T cell receptor (TCR) and CD28 co-activating signals from dendritic cells. iTregs are derived from naive CD4^+^ cells in the peripheral lymphoid tissues when stimulated by appropriate antigens and the existence of TGF-β and IL-2 [67]. Additionally, Foxp3, which is considered as the surface symbolic marker and the most important gene, is a prerequisite for the development and function of Tregs [68]. The NF-κB, NF-κB cofactor IκB NS or Foxo proteins can promote the expression of Foxp3 by combining with regulatory elements at the Foxp3 site [69]. Furthermore, a significant break in the immune blood–spinal cord barrier may make tissues at the lesion site which initially had immune privilege to be recognized and attacked by the peripheral immune system as foreign antigens in the SCI microenvironment. This may facilitate the activation of iTregs.

**Fig. 3:**
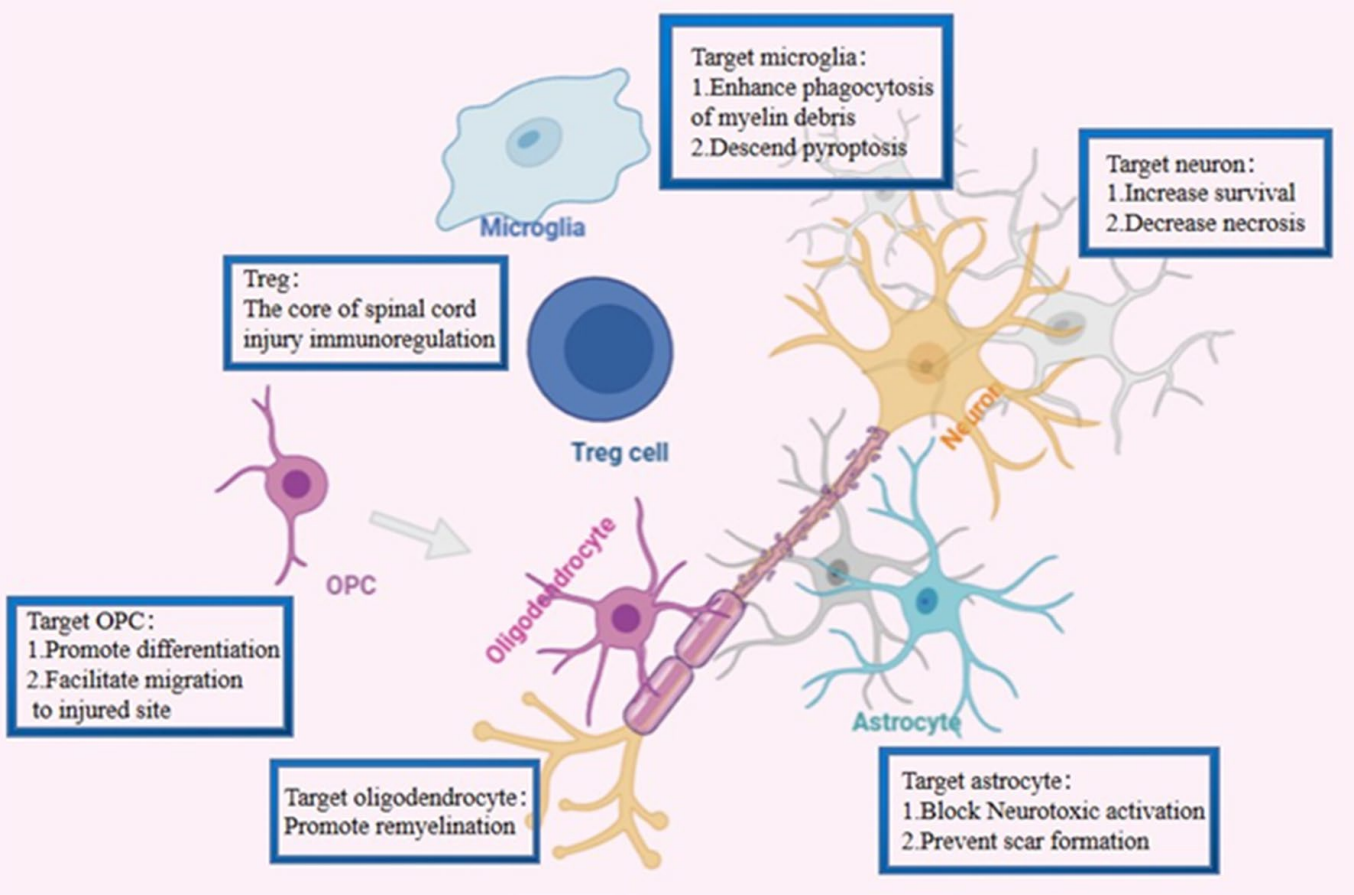
Immunomodulatory effects of Treg cells on in situ cells after spinal cord injury: after spinal cord injury, Treg cells were recruited by the chemokines from the periphery to the site of injury and made a difference. To microglia: Treg can accelerate the clearance of myelin fragments at the site of injury by increasing its phagocytosis, and it can affect the differentiation of microglia by reducing their pyrophosis. To OPC and oligodendrocyte: Treg can influence the migration of OPC to the injured site and promote its differentiation to oligodendrocytes, which in turn promote the remyelination and reduce axon inactivation; To astrocyte: Treg can reduce its neurotoxicity polarization and effectively promote scar formation. To neuron: Treg reverses the microenvironment toward a viable direction and diminishes its necrosis

Several possible mechanisms of Tregs-triggered suppression have been proposed. For instance, Foxp3^+^tregs may play a role in the inflammatory microenvironment of SCI by mediating the secretion of inflammatory cytokines and promoting the anti-inflammatory phenotype of immune cells. Its’ secreted anti-inflammatory cytokines such as IL-10 can further enhance the proinflammatory phenotype dominant balance at the lesion site, which is good for clinical prognosis [70]. Moreover, the reversal from a proinflammatory to an anti-inflammatory environment may improve tissue repair, reduce secondary injured cells, and control the cascade as well as expansion of inflammatory response. The role of Foxp3^+^tregs, however, goes beyond this as they can also kill CTL cells by releasing granzyme B and perforin-1, which cleave and activate endogenous caspases in target cells [71]. Furthermore, Foxp3^+^tregs rob other T cells of IL-2 by expressing the high-affinity IL-2R, hence reducing the amount of proinflammatory immune cells such as Th1 and CD8^+^CTL cells [72, 73]. In fact, Foxp3^+^ Tregs repress neutrophil-driven cytokine secretion in a CD86-dependent manner and TGF-β1 secreted by Foxp3^+^ Tregs facilitates astrocytes differentiation and enhances the generation of tough fibrous tissues at the lesion sites. Foxp3^+^ Tregs also contribute significantly to the control of potential tissue damage in a non-immunological fashion by directly acting on parenchymal cells [71] (Fig. 3). In summary, Foxp3^+^ Tregs may alleviate and regulate secondary spinal cord injury through immune or non-immune approaches. Additionally, they play a positive role in subsequent tissue recovery, although further investigations are still needed to ascertain the specific mechanisms.

### B cells

B lymphocytes, one of the classical immune cells, function in immune defense by releasing antibodies against invasive pathogens. They are derived from hematopoietic stem cells in the bone marrow and migrate to the spleen to promote their maturation and differentiation. B cells can be divided into several subtypes including B-1 cells, B-2 cells, and regulatory B cells, according to the function and gene expression. The B-1 cells are mainly distributed in the abdominal cavity and they can directly recognize low-specificity antigens and produce natural antibodies without antigen presentation by T cells and are, therefore, an important component of innate immunity [74]. The B-2 cells differentiate into plasma cells, characterized by the secretion of antigen-specific antibodies under the stimulation of helper T cells [75]. Furthermore, regulatory B cells which exist in the spleen, lymph nodes, and blood [76] have a strong immunosuppressive activity through the secretion of IL-10, IL-35, and TGF although their proportion in total B cells is relatively low.

High-density antibody labels in the injured spinal cord can detect the accumulation of B cells [77]. Additionally, upregulation of the B cell activation regulator genes, BMCA, APRIL, and BAFF, was observed through microarray analysis of peripheral lymphocyte cells after SCI [78]. As a member of the tumor necrosis factor (TNF) receptor superfamily, expression of BCMA was proven to be positively correlated with the differentiation and activation of B cells [79]. Moreover, BAFF and APRIL are considered as TNF ligands and both can bind BCMA and transcribe B cell survival factors through NF-kB pathways [80]. This, in turn, mediates B cell survival and differentiation into both antibody-secreting plasma cells and long-lived memory B cells [81]. Furthermore, the local release of BAFF as well as APRIL by microglia and astrocytes leads to the establishment and maintenance of B cell number at the injured site after SCI, in the long run. This also contributes to the presence of follicle-like structures near the SCI lesion, hence directing the migration of activated B cells to the lesion.

B cells mediate the process of adaptive immune response to neurotrauma by producing antibodies. In addition, it is thought that SCI alters B cell function both systemically and locally within the spinal cord lesion [82]. According to a recent study, SCI leads to the activation of B cells and production of pathogenic autoantibodies in the spinal cord of mice. Additionally, the nerve function of injured mice without B cells was improved compared to those with normal B cells [77]. Moreover, existing evidence suggests that SCI and the appearance of autoantigens (such as the myelin basic protein and nuclear proteins) lead to the proliferation of B cells and occurrence of IgG autoantibodies in mice [82, 83].The role of autoantibodies after SCI remains largely unclear since the existing suggestions are controversial. For example, one group reported that increased presence of the myelin basic protein autoantibodies after SCI helped in the elimination of myelin debris and that they were not neurotoxic [84]. However, a different report showed that the persistence of autoantibodies prevented the regeneration of neurons over the course of the observation period [85]. A comprehensive study showed that impairment of movement and neuropathy occurred in mice after the injection of antibodies purified from the serum after spinal cord injury. Human studies also detected autoantibodies against the GM1 ganglioside after SCI, and these could inhibit secondary degeneration and promote regeneration [85–87]. Therefore, B cells may repress functional regeneration by releasing anti-GM1 autoantibodies after SCI. Furthermore, based on the overexpression of BCMA, BAFF, and APRIL, it is possible that the autoimmunity induced by SCI promotes the activation of B cells by necrosis debris. These activated B cells, in turn, secrete autoantibodies to induce secondary tissue damage and neurotoxicity after SCI.

## Concluding Remarks

Given the lack of accurate and effective clinical treatment, spinal cord injury has been for the focus of several studies due to the complex pathological events involved. The core event in secondary SCI involves the amplification of inflammatory responses at the lesion site. In addition, the immune system plays an important role in spinal cord injury, since it is the regulatory system of inflammatory response in the human body. The blood–spinal cord barrier can maintain the immune privilege of the spinal cord and prevent the invasion of peripheral pathogens under physiological conditions. However, destruction of the blood–spinal cord barrier may result to the migration and infiltration of peripheral immune cells, which complicate the prognosis of spinal cord injury. Therefore, this review gives a summary of peripheral infiltrating cells and their different roles in the SCI microenvironment. As a result, the review enhances our understanding of the inflammatory environment and the role of each cell in spinal cord injury and highlights possible novel targets for the clinical treatment of SCI.

## Data Availability

Experiment data and materials are available in public databases.

## Abbreviations

SCI: Spinal cord injury
BSCB: Blood–spinal Cord barrier
TJ: Tight junction
CNS: Central nervous system
CXCL: Chemokine (C–X–C motif) ligand
CXCR: Chemokine (C–X–C motif) receptor
CCL: Chemokine (C–C motif) ligand
TNF: Tumor necrosis factor
IL: Interleukin
NO: Nitric oxide
G-CSF: Granulocyte colony stimulating factor
ICAM: Immunoglobulin-like cell adhesion molecule
IKKβ: IκB kinase-β
NF-κB: Nuclear factor kappa-B
VCAM: Vascular cell adhesion molecule
ROS: Reactive oxygen species
SLPI: Secreted leukocyte protease inhibitor
MCP-1: Monocyte chemoattractant protein-1
MIP-1a: Macrophage inflammatory protein 1A
INOS: Inducible nitric oxide synthase
Arg-1: Arginase-1
IFN-: Interferon-γ
TGF-β: Transforming growth factor-β
LPS: Lipopolysaccharide
STAT: Signal transducer and activator of transcription
TLR: Toll-like receptor
DAMP: Damage-associated molecular patterns
COX: Cyclo-oxygen-x
TNFR: Tumor necrosis factor receptor
PPAR: Peroxisome proliferator-activated receptor
KLF: Kruppel-like factor
Msr1: Macrophage scavenger receptor 1
SR-B2: The Class b scavenger receptor
CL-P1: Cleavage factor polyribonucleotide kinase subunit 1
SCARA: The scavenger receptor A
TCR: T cell receptor
APCs: Antigen-presenting cells
PI3K: Phosphatidylinositol 3-kinase
Akt: Protein kinase B
mTOR: Mammalian target of rapamycin
AMPK: AMP-activated protein kinase
HIF-1α: Hypoxia-inducible factor-1α
DC: Dendritic cells
CTLs: Cytolytic T cells
PARP: Poly ADP-ribose polymerase
nTreg: Natural regulatory T cells
iTreg: Induced regulatory T cells
Foxp3: Forkhead box protein 3
BMCA: B Cell maturation antigen
APRIL: A proliferation-inducing ligand
BAFF: B cell activating factor
